# Supplementary material for: A Critical View of the Application of the APEX Software (Aqueous Photochemistry of Environmentally-Occurring Xenobiotics) to Predict Photoreaction Kinetics in Surface Freshwaters
Source: Molecules. 2019 Dec 18;25(1):9. doi: 10.3390/molecules25010009 (PMC7017383; doi:10.3390/molecules25010009)
Supplement: Supplementary file 1 [file molecules-25-00009-s001.zip › 3.2.4_gcc-4.4.0.zip!/3.2.4_gcc-4.4.0/3.2.4_gcc-4.4.0/doc/octave/html/interpreter/User_002ddefined-Data-Types.html]

User-defined Data Types - Untitled


Next: Object Sizes,
Previous: Built-in Data Types,
Up: Data Types


---

### 3.2 User-defined Data Types

Someday I hope to expand this to include a complete description of
Octave's mechanism for managing user-defined data types. Until this
feature is documented here, you will have to make do by reading the code
in the ov.h, ops.h, and related files from Octave's
src directory.
